# High Oxidative Potential Observed in Secondary Organic Aerosol Derived from Oil Sands Emissions

**DOI:** 10.1021/acs.estlett.6c00468

**Published:** 2026-06-10

**Authors:** Qifan Liu, John Liggio, Kun Li, Jeremy J. B. Wentzell, Michael Wheeler, Shao-Meng Li, Colin Lee, Megan He, Lexie Gardner, Drew R. Gentner

**Affiliations:** † School of Environment, State Key Laboratory of Fire Science, 12652 University of Science and Technology of China, Hefei 230026, China; ‡ Beijing National Laboratory for Molecular Sciences (BNLMS), Beijing 100190, China; § Air Quality Research Division, Environment and Climate Change Canada, Toronto, Ontario M3H 5T4, Canada; ∥ Qingdao Key Laboratory for Prevention and Control of Atmospheric Pollution in Coastal Cities, Environment Research Institute, 12589 Shandong University, Qingdao 266237, China; ⊥ State Key Joint Laboratory of Environmental Simulation and Pollution Control, College of Environmental Sciences and Engineering, 12465 Peking University, Beijing 100871, China; # Department of Chemical and Environmental Engineering, 5755 Yale University, New Haven, Connecticut 06511, United States

**Keywords:** secondary organic aerosol, intermediate-volatility organic compounds, oil sands, oxidative potential, atmospheric aging

## Abstract

Oil sands operations in Alberta, Canada, are a large source of secondary organic aerosol (SOA) with unknown health impacts. As a first step in closing this knowledge gap, the oxidative potential (OP), an established metric linking particle exposure to toxicological effects, of oil sands-derived SOA was investigated via OH-initiated oxidation. Experiments used a mixture of gas-phase precursors emitted from oil sands ore that is expected to be representative of mine-face emissions and three relevant single precursors (*n*-decane, *m*-xylene, and naphthalene) for comparison. The OP of the formed SOA in all cases was found to increase rapidly at short simulated photochemical ages (<1.5 days), followed by a decrease upon further oxidation (>2.5 days). This was driven by dynamic changes in SOA composition, particularly organic peroxides, quinones, and unsaturated carbonyls. The measured OP of oil sand ore SOA (OP_OS_) ranged from 38.0 to 60.3 pmol min^–1^ μg^–1^ over photochemical ages of 0.4–4.6 days. This range of OP_OS_ is considerably higher than those reported for other types of aerosol particles typically present in the oil sands region, including the natural background (e.g., α-pinene and isoprene SOA). The results not only provide the first OP measurements of oil sands-derived SOA but also emphasize the importance of accounting for atmospheric aging when evaluating the oxidative potential of SOA.

## Graphical abstract

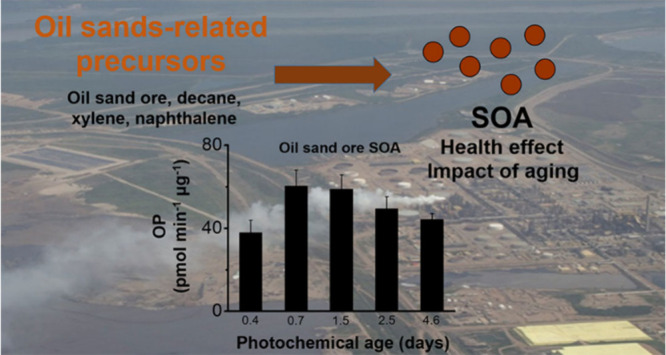

## Introduction

The production of unconventional oil has increased over the past several decades due to the increasing global demand for energy.^1^ The oil sands (OS) in Alberta, Canada are the largest crude oil deposit in North America extracted by unconventional means, with oil reserves of 1.7 trillion barrels.^2^ However, the large-scale oil production in the oil sands region has raised substantial environmental concerns associated with pollutant release.^3–6^

Recent evidence suggests that oil sands operations are a large source of secondary organic aerosol (SOA) formed through atmospheric oxidation of gas-phase organic precursors, which leads to estimated atmospheric SOA formation rates of 45–84 tons day^–1^.^2^ Such large SOA formation rates results in SOA constituting a sizable fraction of PM_2.5_ (particulate matter with diameter less than 2.5 μm) in the OS region.^2,7^ Consequently, extensive efforts have been devoted to characterizing the sources and chemical composition of SOA in OS region.^8–11^ Despite progress in understanding its physicochemical properties,^9–11^ and in identifying its existence during high pollution events in local OS communities,^12^ information on the human health impacts of OS-derived SOA remains lacking. Given that numerous studies have shown strong associations between human exposure to PM (including SOA) and various detrimental health outcomes (e.g., cardiovascular and respiratory diseases),^13–15^ the investigation of the potential for health effects from OS-derived SOA is especially important.

A well-known chemical composition-dependent biochemical mechanism that may contribute to PM toxicity is the cellular oxidative stress pathway, induced by PM-bound redox-active constituents (e.g., peroxides and quinones).^16^ Specifically, the inhalation of PM can cause the depletion of antioxidants (e.g., glutathione), leading to the perturbation of the cellular redox balance and cell damage via the formation of reactive oxygen species.^17^ Oxidative potential (OP), defined as the ability of PM to oxidize antioxidants, is now considered a key metric linking PM exposure to its toxicological effects (e.g., acute cardiorespiratory end points).^17–22^ One of the most widely used OP measurement techniques is the dithiothreitol (DTT) assay, which is known to be sensitive to organic compounds.^17^ Therefore, quantifying the OP^DTT^ of oil sands-derived SOA would provide valuable information for assessing the potential health risks associated with PM exposure in the OS region.

Here, the OP of four types of SOA relevant to the oil sands were investigated. This includes SOA derived from the emissions of gas-phase precursors from recently mined (unprocessed) oil sands ore, and from three single relevant precursors known to be emitted from OS operations (*n*-decane, *m*-xylene, and naphthalene; spanning alkanes, single ring-aromatics, and polycyclic aromatic hydrocarbons, respectively). Combining the OP measurements with simultaneous analysis of SOA oxidation state and organic peroxide content (a potential driver of OP) across a range of photochemical ages, provides important insights into the sources of total PM_2.5_-associated OP for the OS region, its evolution over time and space, and its relative impact compared to other non-OS sources.

## Materials and Methods

### Generation of SOA

The OH radical initiated oxidation of unprocessed oil sands ore evaporative emissions and three types of relevant precursors (*n*-decane, *m*-xylene, and naphthalene; used for comparison) were performed using the Environment and Climate Change Canada oxidation flow reactor (ECCC-OFR).^10^ Vapors from the precursors were introduced into the OFR by a small flow of zero air (0.5–1 mL min^–1^) passing over the headspace of the sample material, which was placed in a U-shaped glass tube. The mixing ratios of gaseous precursors (270–450 ppbC) were measured using a total hydrocarbon method,^10^ with details given in the Supporting Information (SI) Text S1. OH radicals were generated via UV photolysis (254 nm) of ozone (2 ppm) with water vapor (50% RH) at 298 K. In offline calibrations, the OH exposure was calculated through the decay of CO gas inside the OFR,^23^ and was in the range of 1.56 × 10^11^–1.98 × 10^12^ molecules cm^–3^ s. This experimental exposure corresponds to 0.4–4.6 days of photochemical age (under low-NO_*x*_ conditions), commensurate with the OH concentrations estimated in highly oxidative OS plumes containing these precursors (average daytime summer OH = 1 × 10^7^ molecules cm^–3^; see Figure S1).^24^ The composition of the oil sands ore emissions was analyzed offline using gas chromatography followed by both an electron ionization mass spectrometer and an atmospheric pressure chemical ionization high-resolution time-of-flight mass spectrometer (GC-EI-MS and GC-APCI-TOF),^25,26^ with details given in SI Text S2. SOA chemical composition was measured using a HR-TOF-AMS (high-resolution time-of-flight aerosol mass spectrometer) and an EESI-TOFMS (electrospray ionization time-of-flight mass spectrometer).^27,28^ Further details regarding SOA sample collection and extraction are given in SI Text S1.

### Oxidative Potential Measurement

The DTT assay was employed to quantify the oxidative potential of SOA, as described in detail in previous studies.^29,30^ Briefly, 200 μL of DTT (0.5 mM in phosphate buffer), 700 μL of particle extraction solution (*n* = 3), and 100 μL of potassium phosphate buffer were added to a glass vial. The estimated concentration of particle solution was 40–76 μg/mL. The reaction mixtures were incubated at 310 K for 45 min, at which point the reactions were quenched by 1 mL of 5–5′-dithiobis (2-nitrobenzoic acid) (DTNB; 1 mM). The absorbance of 5-thio-2-nitrobenzoic acid (TNB) formed by the reaction of DTNB with residual DTT was measured at 412 nm using a UV–VIS spectrophotometer (Ocean Optics). The mass-normalized DTT consumption rate (OP; pmol min^–1^ μg^–1^) can be calculated based on the TNB measurements, reaction time, and the sample mass (SI Text S3).

### Total Peroxide Measurement

Peroxides are a known trigger of OP in PM and were quantified as total peroxides in the SOA using an iodometric–spectrophotometric method,^31^ with details provided in SI Text S4. To estimate the contribution of peroxides to the OP of SOA (C_p_; %), we assume that the peroxides present in the SOA (most of which lack available standards) possess the same DTT reactivity as benzoyl peroxide (a commercially available peroxide; see calculation details in SI Text S4). This assumption has been used in previous analysis of peroxides in polycyclic aromatic hydrocarbon-derived SOA,^32^ due to the structural similarity between benzoyl peroxide and those formed during oxidation.

## Results and Discussion

### Dynamic Changes in the OP of Oil Sands-Related SOA during Aging

The measured OP of OS ore-derived SOA (OP_OS_) and those from single precursors are shown in Figure 1A as a function of photochemical age. On average, OP_OS_ is considerably lower than the OP of naphthalene-SOA (OP_NAH_), but is up to 4 times higher than the OP of decane-SOA (OP_DEC_) and *m*-xylene-SOA (OP_XYL_), i.e., OP_NAH_ > OP_OS_ > OP_XYL_> OP_DEC_. Regardless of the absolute magnitude of the OP, a similar maxima in OP was observed for all investigated SOA types as a function of photochemical age (Figure 1A). For example, during the initial OH exposure, the OP_OS_ increased by 59%, from (38.0 ± 5.85) to (60.3 ± 7.79) pmol min^–1^ μg^–1^, with increasing photochemical age (0.4–0.7 days), followed by decreases in OP (44.3–49.4 pmol min^–1^ μg^–1^) with further photochemical aging (2.5–4.6 days).

**Figure 1:**
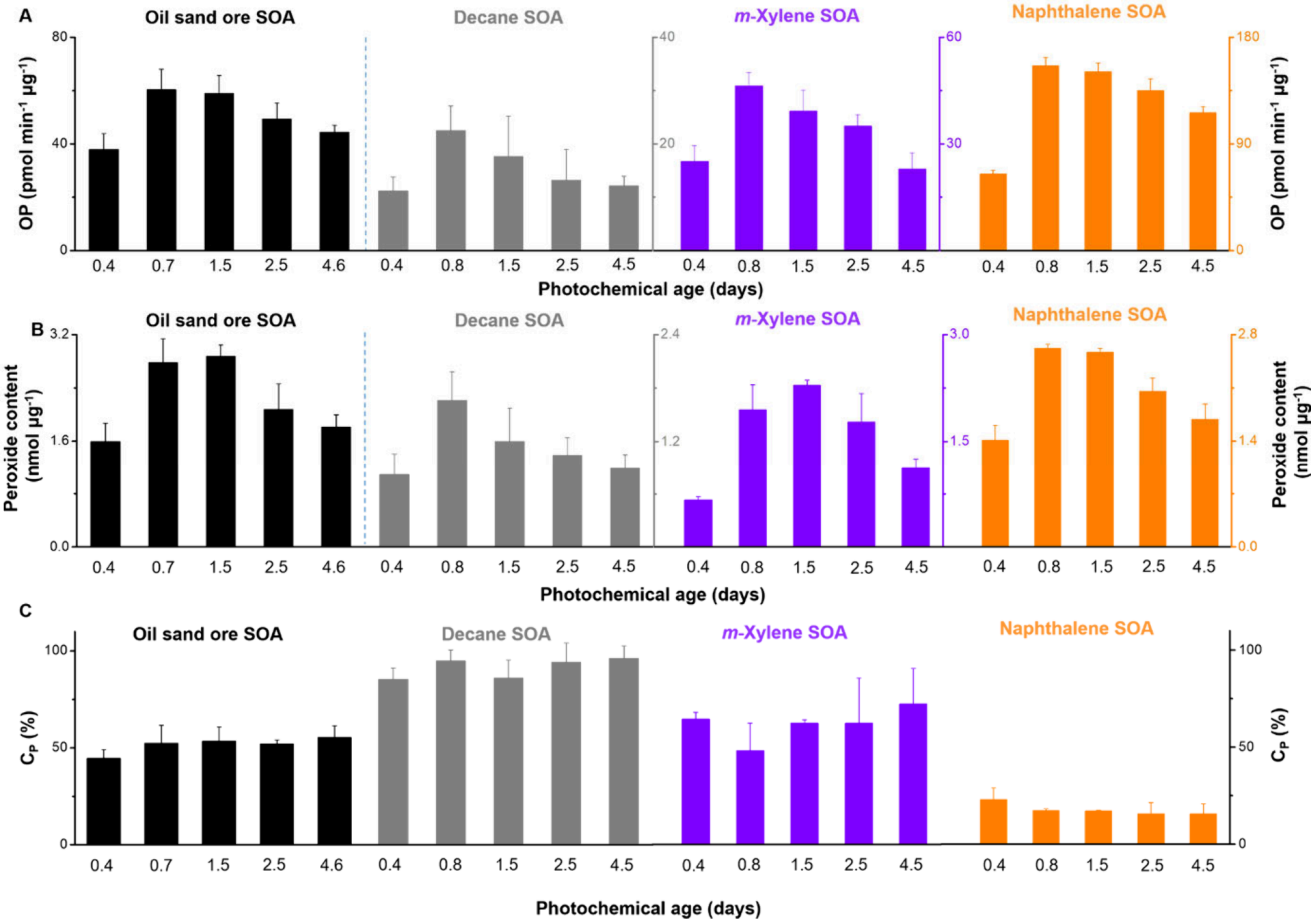
(A) Oxidative potential (OP; pmol min^–1^ μg^–1^) and (B) peroxide content (nmol μg^–1^) of SOA (from precursors including an oil sand ore mixture off-gassing, *n*-decane, *m*-xylene, and naphthalene) measured as a function of photochemical age. (C) The estimated contribution of peroxides (C_p_; %) to the OP of different types of SOA.

Elucidating the cause of the OP relationship with photochemical age requires inquiry into the chemical evolution of the SOA during photo-oxidation. Figure 2A shows the bulk aerosol elemental ratios for the four types of SOA investigated at different photochemical ages, which provide information on the degree of SOA functionalization. The observed SOA spans a wide range of O/C and H/C ratios. For example, the O/C ratio of oil sands ore-derived SOA increased from 0.46 ± 0.03 to 0.85 ± 0.10, with an increase in photochemical age from 0.4 to 4.6 days. The measured Δ­(H/C)/Δ­(O/C) slopes for oil sands-SOA, decane-SOA, and *m*-xylene-SOA ranged from – 0.60 to – 0.35, suggesting OH addition or hydrogen abstraction followed by addition of oxygenated functional groups (e.g., hydroxycarbonyls and peroxides) and also fragmentation reactions that lead to loss of carbon and hydrogen from SOA at longer photochemical ages.^33^ As shown in Figure 2B, the oil sand ore off-gassing had a mixture of aromatics, alkanes, and alkenes, with a large fraction of intermediate-volatility and semivolatility organic compounds (IVOCs and SVOCs). Such an observation is similar to prior work and the understanding that I/SVOCs are dominant contributors to the formation of oil sands-related SOA.^2,9^ The chemical nature of oil sands ore emissions (i.e., a complex mixture of aromatics, alkanes, and alkenes) results in the formed SOA possessing a similar elemental ratio evolution pattern as some other common types of SOA (e.g., alkane and single ring-aromatic SOA in Figure 2A) during photo-oxidation. In contrast, the H/C ratio of naphthalene-SOA remained relatively constant during SOA aging, which is consistent with previous findings.^34,35^ This may result from OH addition and/or from the condensation of oxidized products that maintain the H/C ratio.

**Figure 2:**
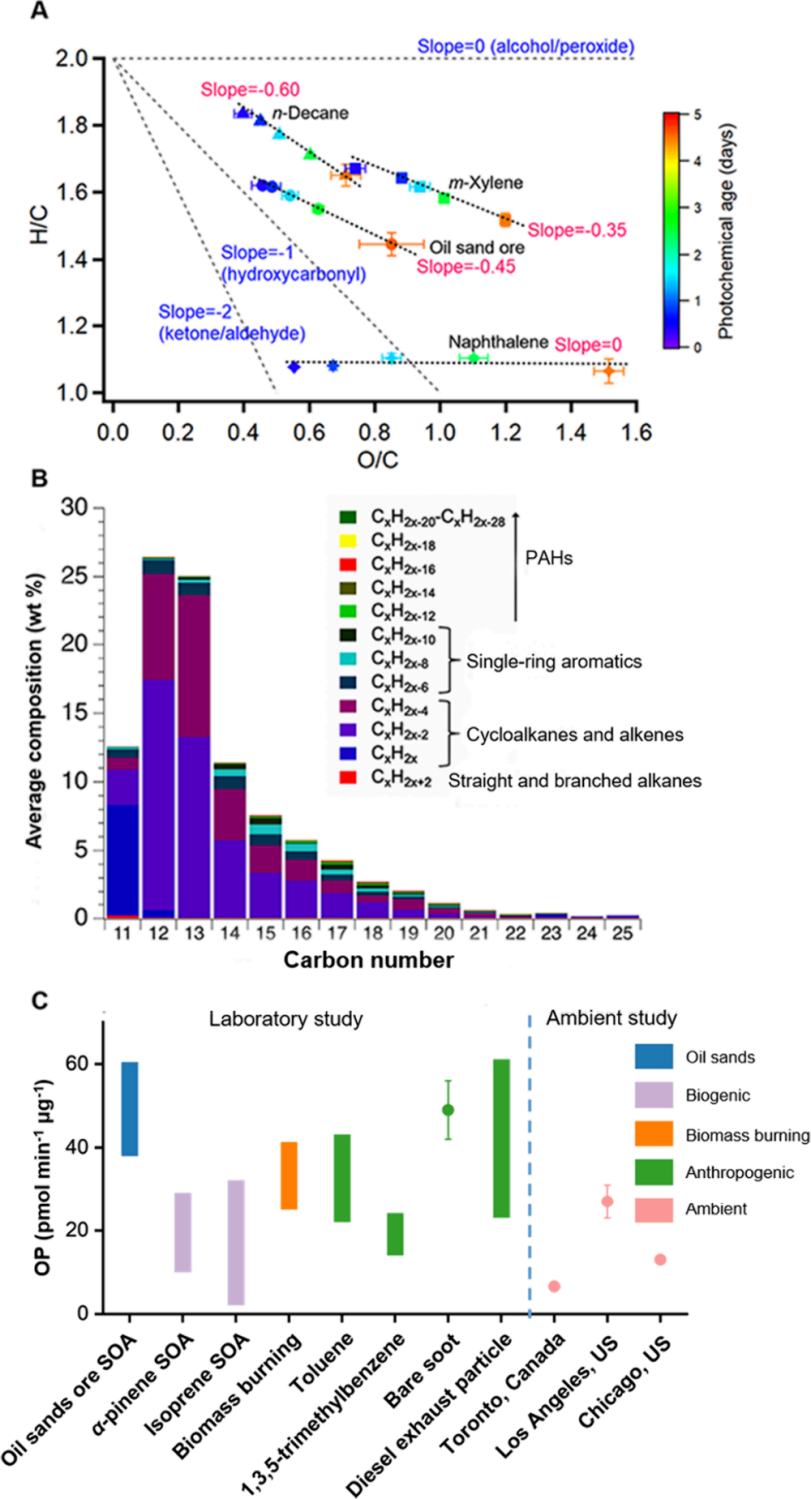
(A) Van Krevelen diagram for SOA formed from OH-initiated oxidation of oil sands-related precursors (unprocessed oil sand ore vapor emissions, *n*-decane, *m*-xylene, and naphthalene). The photochemical ages are calculated assuming an average daytime summer OH concentration in OS plumes (1 × 10^7^ molecules cm^–3^).^24^ (B) Intermediate (C12–C18) and semivolatility (C19–C25) fractional composition of oil sands ore-derived emissions used in SOA experiments. (C) Oxidative potential of various emission sources studied in chamber or ambient conditions (OP were determined using the DTT assay under similar experimental conditions). Data source: Oil sands ore SOA (this study), α-pinene SOA,^21,36^ isoprene SOA,^29,36^ fresh and aged wood smoke,^36–38^ toluene and 1,3,5-trimethylbenzene SOA,^36^ bare soot,^39^ diesel exhaust particle,^30,40^ ambient particles in Toronto,^30^ Canada; Los Angeles, US;^41^ and Chicago, US.^42^ OP data are shown as bars if different OP values were reported for the same type of PM, or shown as data points for single-value reports in the literature.

In addition to the aerosol elemental ratio analysis, we further investigate the evolution of organic peroxide content during SOA aging. Previous studies suggest that organic peroxides could be important contributors to the OP of SOA as they can oxidize DTT to disulfides or other sulfur-containing compounds, leading to its depletion, as shown in Figure S2.^20,43^ The measured peroxide content of the four types of SOA investigated in Figure 1B exhibited similar trends as their OP in Figure 1A across the investigated photochemical age range (i.e., peroxide content increased at low photochemical ages and then decreased with further photochemical oxidation), with maximum peroxide contentat photochemical age of 0.7–1.5 days. The concurrent changes observed between the total peroxide content and the measured OP implies that peroxides were partly responsible for changes in OP. The SOA peroxide content is governed by two key processes, including the formation of peroxides through HO_2_ + RO_2_ reactions versus the degradation of peroxides through photolysis and reactions with other particulate compounds (e.g., carbonyls; see Figure S3).^44,45^ The enhancement of peroxide content in the early stage of SOA aging (<1.5 days; Figure 1B) is likely ascribed to the enhanced formation of peroxides with increased OH exposure. Conversely, the decrease of peroxide content at high photochemical ages (>2.5 days) suggests that peroxide degradation processes dominate over new formation.

While the resultant changes in peroxide content can partly explain the changes in OP across the photochemical ages investigated (Figure 1A and B), they do not account for the overall OP. As shown in Figure 1C, the fractional peroxide contributions to the OP of SOA (C_p_) were estimated to be 14–96%, depending upon the SOA type and photochemical ages. Specifically, the peroxide contribution to OP was largest for decane-SOA (85–96%) and smallest for naphthalene-SOA (14–23%), with that of OS ore-derived SOA and xylene-SOA falling between these values (44–72%). This suggests that other nonperoxide compounds also contributed to the OP across the SOA types investigated. Based upon known atmospheric precursor oxidation mechanisms and the principles of redox reactions (e.g., previously reported chemical–DTT reaction mechanism), additional potential OP contributors to SOA (under low NO_*x*_ conditions) may include quinones and unsaturated carbonyls.^32,43^ Quinones can be formed through OH reaction with aromatics such as polycyclic aromatic hydrocarbons (Figure S4),^46^ which can catalytically oxidize DTT through a redox cycling, as in the case of 1,4-naphthoquinone (Figure S2). Unsaturated carbonyls can be formed via ring-opening reactions during OH-initiated photo-oxidation of aromatics (Figure S4), which can also consume DTT through nucleophilic addition reactions (Figure S2).^47^

Accordingly, the C_p_ of naphthalene SOA (Figure 1C) was small, which was less than 23% across the investigated photochemical ages. This is consistent with the fact that quinones (e.g., 1,4-naphthoquinone) make an important contribution to OP_NAH_, which are known to be formed and are highly DTT active.^48^ As well, unsaturated carbonyls can contribute to OP_NAH_. This is further supported by EESI-TOF-MS measurements of the naphthalene SOA, which demonstrated that the specific signal intensities of C_10_H_6_O_2_ (likely attributed to a naphthoquinone) and C_10_H_8_O_2_ (likely attributed to an unsaturated carbonyl) were well correlated with the observed OP_NAH_ across the investigated photochemical ages (Figure S5). In the case of *m*-xylene SOA, the C_p_ ranged from 48% to 72%, depending upon the photochemical age, which highlights the essential role of peroxides in determining the OP of *m*-xylene SOA. Given the known low formation yield of quinones (yield <5%) and high formation yield of dicarbonyls (yield = 25%; many of which are unsaturated) from photo-oxidation of *m*-xylene,^49^ unsaturated carbonyls may be the other key contributor to its OP. With respect to decane-SOA, its high C_p_ (>85%) indicates that peroxides are the dominant source of OP_DEC_. This is in agreement with known alkane oxidation mechanisms, which may produce a very small fraction of unsaturated carbonyls and cannot form quinones during oxidation.^50,51^

In the case of OS SOA, the estimated fractional peroxide contribution to the OP was 45–55%, highlighting the important role of nonperoxide contributors in determining OP_OS_. As shown in Figure 2B, alkenes, cycloalkanes, and single ring-aromatics make up a large fraction of the OS ore emissions. Given the OH oxidation mechanism for these compounds, it is unlikely that high concentration of quinones are formed during oxidation.^49–51^ In this case, unsaturated carbonyls, potentially formed from oxidation of alkenes and single ring-aromatics, can be an important nonperoxide contributor to OP_OS_. Indeed, previous studies indicated that unsaturated carbonyls can be formed as major products during OH oxidation of some alkenes and single ring-aromatics.^52,53^ We also observed the formation of potential unsaturated carbonyls (e.g., C_12_H_22_O) from OS ore oxidation, for which its signal intensity shows a similar evolution pattern with the OP_OS_ across the investigated photochemical ages (Figure S6). Regardless, further studies are warranted to investigate the impact of OS-related alkene and aromatic photochemistry on the OP of SOA. In addition, given the chemical nature of oil sands ore emissions (a mixture of alkenes, alkanes, and aromatics), it is not surprising that its OP exhibits a similar trend as those of single precursors across the investigated photochemical ages (Figure 1C).

### Comparison to Other Types of PM

The results provide important insights into the potential health impacts associated with SOA formed from oil sands-related precursor mixtures, especially when placed in context with the DTT activity of other relevant source types and locations (conducted under similar experimental DTT concentrations and reaction conditions), as shown in Figure 2C. The mass-normalized OP of oil sand ore-related SOA reported here (38–60 pmol min^–1^ μg^–1^) is comparable to the OP of diesel combustion exhaust particles (23–61 pmol min^–1^ μg^–1^).^30,40^ However, despite similar OP per unit of PM mass, diesel combustion exhaust emissions make only minor contributions to the regional I/SVOC burden downwind of oil sands operations or to the formation of PM_2.5_ in OS region.^2,9^ Conversely, noncombustion-related I/SVOCs as investigated here, contribute disproportionately to the total organic carbon in oil sands plumes (up to 60%),^2^ suggesting that the OP for noncombustion-related SOA in downwind oil sands plumes may be a dominant source of OP for PM_2.5_ in the region. The OP for OS ore SOA is also higher than the OP of other types of PM including biogenic SOA (α-pinene and isoprene SOA; < 35 pmol min^–1^ μg^–1^)^21,29,36^ and fresh and photochemically aged biomass burning PM (25–41 pmol min^–1^ μg^–1^),^36,37^ which influence the region. Comparisons between the OP of lab-generated SOA and those from other ambient region is challenging because the sources of PM in urban areas and hence their combined contributions to OP are more varied. Nonetheless, the OP of oil sand ore SOA is higher than those of ambient fine particles in highly populated North American cities (Figure 2C) such as Toronto, Los Angeles, and Chicago (OP = 6.6–27 pmol min^–1^ μg^–1^).^30,41,42^ While not a direct comparison, the elevated OP of oil sands ore SOA (relative to PM in urban areas) implies that during events where OS-derived SOA dominates PM mass,^2^ the associated OP could be large compared to the OP from a more varied mixture of SOA types in typical urban centers. This highlights the need for ambient OP measurements, to provide further constraints on the potential health risk of PM in the oil sands region.

### Implications

An important motivation of many oil sands-related investigations is understanding the impacts of OS emissions on local communities within and around the oil sands region. Previous studies on this topic have mainly focused on mass concentration measurements of specific air pollutants (e.g., PM_2.5_), which were reported to be low in the main population centers of the region (<10 μg m^–3^), and suggested minimal adverse health effects from OS activities.^54,55^ However, recent studies have indicated that PM chemical composition plays an equally important role to PM mass in driving negative health effects,^38,56–58^ and have found positive associations between mortality in the general population and exposure to PM_2.5_ below the US National Ambient Air Quality Standards.^59^ Community exposure to PM_2.5_ in the vicinity of the oil sands is impacted by contributions from both OS-related sources and non-OS sources (e.g., biogenic SOA and biomass burning PM). Considering that OS-derived SOA can make up a large portion (>75%) of the organic PM in local oil sands communities in the absence of wildfire smoke,^2,12^ and the higher OP of OS ore-SOA compared to biogenic SOA (Figure 2C), OS-derived SOA likely plays a key role in determining the oxidation potential of inhaled PM in communities during nonsmoke impacted periods. The results imply that the potential health effects of OS-derived SOA should be taken into account when assessing the exposure risks of PM_2.5_ in these communities in addition to more traditional PM mass metrics. This is particularly important given that studies on the health impacts of OS-related air pollutants are rare.^60^

The current results also indicate that the photochemical aging of SOA is an additional factor that must be considered when assessing the OP of SOA, as freshly formed SOA (photochemical age <1.5 days) particularly from oil sands-related emission sources is likely to be more OP-active than highly aged SOA (photochemical age >2.5 days). The maxima in the OP of SOA is not the same across all compound classes and sources,^37,38^ which highlights the importance of improved oxidation mechanism understanding for determining the compositional differences that arise within SOA and give rise to OP. More broadly, this work underscores the importance of photochemical aging as a major determinant of the magnitude of OP of SOA, not only in the oil sands, but across other anthropogenic-related sources. Therefore, accounting for such processes will be especially important for making accurate model predictions of OP associated health risks, which are increasingly being used.^56^

This is the first study to report the OP of oil sands SOA, which is subject to uncertainties and will require further investigation. Key areas of future work include the determination of OP across a more diverse array of oil sands sources including tailings ponds and their associated SOA formed in the presence of NO_*x*_ (see further discussion in Text S5).^61^

## Supplementary Material



## Notes

OFR experiments, oil sands ore off-gassing emission characterization, OP measurements, total peroxide measurements, SOA composition analysis, future perspectives, estimated OH concentrations in OS plumes (Figure S1), reaction mechanism between DTT and organics (Figure S2), formation and degradation mechanisms of peroxides (Figure S3), formation mechanism of redox-active constituents during oxidation (Figure S4), signal intensities of naphthoquinones and unsaturated carbonyls (Figures S5 and S6), and calibration curves and DTT reactivity for peroxides (Figure S7) (PDF)
