# Supplementary material for: High Oxidative Potential Observed in Secondary Organic Aerosol Derived from Oil Sands Emissions
Source: Environ Sci Technol Lett. 2026 Jun 10;13(7):953–60. doi: 10.1021/acs.estlett.6c00468 (PMC13374638; doi:10.1021/acs.estlett.6c00468)
Supplement: Supplementary file 1 [file ez6c00468_si_001.pdf]

Supporting Information for

High Oxidative Potential Observed in Secondary Organic Aerosol Derived from Oil Sands Emissions

Qifan Liu[1,2]*, John Liggio[3]*, Kun Li[4], Jeremy J. B. Wentzell[3], Michael Wheeler[3], Shao-Meng Li[5], Colin Lee[3], Megan He[6], Lexie Gardner[6], Drew R. Gentner[6]

1. School of Environment, State Key Laboratory of Fire Science, University of Science and Technology of China, Hefei 230026, China

2. Beijing National Laboratory for Molecular Sciences (BNLMS), Beijing 100190, China

3. Air Quality Research Division, Environment and Climate Change Canada, Toronto, Ontario M3H 5T4, Canada

4. Qingdao Key Laboratory for Prevention and Control of Atmospheric Pollution in Coastal Cities, Environment Research Institute, Shandong University, Qingdao 266237, China

5. State Key Joint Laboratory of Environmental Simulation and Pollution Control, College of Environmental Sciences and Engineering, Peking University, Beijing 100871, China

6. Department of Chemical and Environmental Engineering, Yale University, New Haven, Connecticut 06511, United States

*Corresponding authors.

Email: liuqifan@ustc.edu.cn (Q.L.); john.liggio@ec.gc.ca (J.L.).

(17 Pages, 7 Figures)

Contents

S1. OFR experiments and sample analysis: Page S3

S2. Oil sands ore off-gassing emissions characterization: Page S4

S3. Oxidative potential measurement: Page S5

S4. Total peroxide measurement and C_p calculation: Page S6

S5. Futhure perspectives: Page S7

S6. Modelled OH in oil sands plumes for OFR photochemical age determination: Page S8

S7. Further discussion on SOA composition and OP of SOA: Page S9

Figures

S1: Estimated OH concentrations in OS plumes.

S2: Reaction mechanism between DTT and organics in SOA.

S3: Formation and degradation mechanisms for peroxides.

S4: Formation mechanisms of peroxides, quinones, and unsaturated carbonyls during OH oxidation.

S5: Signal intensities of naphthoquinones and unsaturated carbonyls for naphthalene SOA.

S6: Signal intensities of unsaturated carbonyls for *m*-xylene and OS ore SOA.

S7: Calibration curves and DTT reactivity for peroxides.

SI Text

S1. OFR experiments and sample analysis

Reagents

Mined oil sands ore samples were provided through the Canada's Oil Sands Innovation Alliance (COSIA), while *n*-decane (# 457116), *m*-xylene (# 296325), naphthalene (# 147141), methanol (# 646377), dithiothreitol (# D0632), 5-5'-dithiobis (2-nitrobenzoic acid) (# D8130), 1,4-naphthoquinone (# 152757), potassium iodide (# 60399), benzoyl peroxide (# 179981), hydrogen peroxide (30 wt% in H_2O, #H1009), acetic acid (# 695092), and cumene hydroperoxide (# 247502) were purchased from Sigma-Aldrich. CO gas (0.1% in air) was purchased from Air Liquide. O_3 was produced by an O_3 generator (Ozone Solutions, model TG-10) supplied with high purity O_2 (Air Liquide, 99.999%).

Gaseous precursor measurement and SOA formation

The total flow rate inside the OFR was 6 L min^{-1}. Vapors derived from the unprocessed ore are expected to represent emissions downwind of the surface mining operations primarily, but are likely to be compositionally similar to other facility hydrocarbon emissions, given the known similarities in volatility distributions of emissions across OS sources. The mixing ratios of gaseous precursors were determined using a total hydrocarbon approach. In offline experiments, the total hydrocarbon (THC) concentration entering the ECCC-OFR was determined by passing the input gas stream through a Pt-based catalytic converter maintained at 400 degrees and measuring the subsequently evolved CO_2 (LI-COR LI-840A).[1] The conversion efficiency of this THC system was reported to be ~100% for several hydrocarbons in the range of C7–C18. The evolved CO_2 mixing ratio (ppb) is converted to a total carbon mixing ratio (ppbC). With the measured total carbon mixing ratio and the carbon number of the individual precursors (C10, C8, and C10 for *n*-decane, *m*-xylene, and naphthalene, respectively), we can determine their mixing ratios. Previous work suggested that the average carbon number of oil sand ore precursors was ~C10.[1] With this CO_2-to-carbon convention, the mixing ratios of gaseous precursors were estimated to be 270–450 ppbC. The estimated mixing ratios correspond to 27–45 ppb for the gaseous precursors assuming a C10 molecule. The concentrations are within the concentration range observed in OS regions (up to 1 ppmC) and are also within the concentration range used in previous OFR and smog chamber studies.[1-3] SOA was formed through OH oxidation of gaseous precursors without the presence of seed particles. The SOA mass concentration (26–125 μg m^{-3} for different types of SOA at different photochemical ages) was calculated by multiplying the integrated volume concentration from the SMPS (scanning mobility particle sizer) by the effective particle density. The effective density (ρ =1.35–1.51 for different types of SOA) was calculated based on the vacuum aerodynamic diameter (D_{va}; obtained from the AMS) and the electric mobility diameter (D_m; obtained from the SMPS) using

the equation ($\rho = D_{va}/D_m$). EESI-TOFMS (electrospray ionization time-of-flight mass spectrometer), which can provide online molecular information for SOA, was also used to analyze SOA chemical composition.[4]

Aerosol samples were collected on 47 mm Teflon filters (Savillex) at the exit of the OFR for 1–2 h with a flow rate of 5 L min^{-1} with oxidants removed via an activated carbon denuder during the sampling time. For OP measurements, filter samples were extracted in 5 mL HPLC-grade methanol by sonication at room temperature. To avoid overheating and to maintain a consistent extraction temperature, the ultrasonic bath chamber was supplemented with an ice-water mixture, which kept the bath temperature at room temperature. To evaluate the potential impact of extraction procedure on SOA composition, we examined the recoveries of benzoyl peroxide, cinnamaldehyde, and 1,4-naphthoquinone, which represent peroxides, unsaturated carbonyls, and quinones, respectively. The recoveries of these chemicals were in the range of 87–95%. This demonstrates that extraction would not to lead significant changes in SOA composition, particularly redox-active compounds, which are of concern here. The particle extracts were then concentrated to 1 mL under a gentle nitrogen stream. Sample extracts were filtered using a PTFE filter (0.22 μm pore size; LabExact). Total mass collected was determined based on the SOA mass concentration and the total volume of air collected.

OH exposure measurement

The OH exposure (molecules cm^{-3} s), which is the product of OH concentration (molecules cm^{-3}) and residence time (s) in the reactor, was determined by measuring the loss of CO as a function of O_3 concentration in offline calibrations:[5]

$$\text{OH exposure} = -\frac{1}{k_{\text{CO}}}\ln\frac{[\text{CO}]_t}{[\text{CO}]_0} \tag{S1}$$

where k_{CO} is the second-order rate constant of CO at 298 K (1.54×10^{-13} cm^3 $molecules^{-1}$ s^{-1}),[6] $[CO]_t$ is the CO concentration measured by a CO monitor (LGR, model 23r) in the presence of O_3, $[CO]_0$ (1.5 ppm) is the measured CO concentration in the absence of O_3. The measured OH exposures ranged from 1.56×10^{11} to 1.98×10^{12} molecules cm^{-3} s. Assuming an average daytime summer OH concentration (1.0×10^7 molecules cm^{-3}; Text S6 and Figure S1) in highly oxidative OS plumes,[7] this experimental exposure is equivalent to 0.4−4.6 days of atmospheric photochemical oxidation.

S2. Oil sands ore off-gassing emissions characterization

The composition of the oil sands ore off-gassing emissions used to form SOA in the OFR was analyzed through a bench-scale experiment with replicate samples collected over the course of one day. Approximately 150 g of ore sample was transferred to a precleaned 3.8 L glass vessel and arranged in a single layer to maximize surface area exposure. Temperature was controlled

by wrapping the glass vessel in aluminum foil and placing it in a GC oven (Varian) held at 25 ℃. A thermocouple (Omega) attached to the side of the container and a laser temperature reader (Ames Instruments) were used to monitor the temperature of the ore. Atmospherically relevant air exchange rates were achieved using a constant flow of zero air (Aadco zero air generator) at 5.0 SLPM (Alicat mass flow controller) into the sample vessel. Samples with replicates were collected using adsorbent tubes similar to prior work at multiple time points throughout the first day and analyzed offline using GC-EI-MS and GC-APCI (Gerstel TD).[2,8,9] A ~4 in. 316 stainless steel sampling line, heated to 45 ℃ to reduce losses to the tubing material, was used at the outlet of the sample vessel for sample collection. A detailed description of the methodology and adsorbent tube sample collection and analysis can be found in the SI of He et al. as the ore off-gassing experiment here used the same equipment and methodology as the mature fine tailings (MFT) off-gassing experiments.[2]

S3. Oxidative potential measurement

Dithiothreitol (DTT) assay is one of the most commonly used OP measurement techniques, which have been used in numerous PM studies. [10-12] Given this, DTT assay was employed in this study to quantify the oxidative potential of SOA using the aerosol samples described above. The reaction between 5-5'-dithiobis (2-nitrobenzoic acid) (DTNB) and DTT resulted in the production of 5-thio-2-nitrobenzoic acid (TNB) which was quantified by its absorbance at 412 nm using a UV-VIS spectrophotometer. The mass normalized DTT consumption rate (OP; pmol min^{-1} μg^{-1}) can be calculated using Eq. S2 and Eq. S3:[13]

$$\Delta \mathrm{DTT} = \frac{\mathrm{A_b} - \mathrm{A_s}}{\mathrm{A_0}} \mathrm{DTT_0} \qquad \text{(S2)}$$

$$\mathrm{OP} = \frac{\Delta \mathrm{DTT}}{\mathrm{m} \times \mathrm{t}} \qquad \text{(S3)}$$

where ΔDTT is blank-corrected DTT consumption (nmol) by the aerosol sample, DTT_0 is the initial moles of DTT (100 nmol), A_0 is the initial absorbance of the blank sample, A_s is the absorbance of the aerosol sample after interacting with DTT for a certain time (45 min in this study), A_b is the absorbance of the blank sample over the same period of time, m is the mass of aerosol sample (μg), and t is the interaction time (45 min). There is a 3% loss of DTT in the blank samples after interaction for 45 min.

The experimental procedures associated with DTT analysis are slightly different across the available literature. It was found that a key factor that impacts the DTT activity measurement results is the initial DTT concentration.[14] Therefore, to evaluate the relative impact of oil sands SOA relative to other types of PM, we compare the currently measured OP (initial DTT

concentration 100 μM, temperature 37 °C, pH 7.4) with those reported for other types of PM in previous literature (conducted at similar experimental conditions).

S4. Total peroxide measurement and C_P calculation

Quantification of peroxides

The quantification of peroxides in SOA was conducted using an iodometric–spectrophotometric method which results in the reactions: $ROOH + 2I^- + 2H^+ \rightarrow ROH + H_2O + I_2$; $I_2 + I^- \rightarrow I_3^-$; where I^- is first oxidized to I_2 by peroxides under acidic conditions, and then I_2 reacts with the remaining I^- to form I_3^-, a compound that can be detected spectrophotometrically.[15] In this approach, 500 μL of the SOA extract (*n*=3) was mixed with 100 μL acetic acid solution (1.4 M) and purged with a flow of 20 sccm of nitrogen for 2 min to exclude dissolved oxygen. An additional 300 μL KI (180 mM) was added to the subsequent solution, and the vial was capped immediately and slightly pressurized with nitrogen. For the filter blank value determination, no KI was added to the filter extract. Extractions vials were then placed in an orbital shaker (Benchmark Scientific; model H1001-M) at 37°C and 100 rpm. After 1 h of reaction, the absorbance was measured with a UV-VIS spectrophotometer (Ocean Optics; model HR4000CG-UV-NIR) at λ=351 nm. After testing for the sensitivities of various peroxides (benzoyl peroxide, hydrogen peroxide, and cumene hydroperoxide) in the KI assay (Figure S6A), benzoyl peroxide was chosen to represent peroxides in SOA and used as a standard for calibration, which is consistent with previous studies on peroxides.[16,17] The peroxide content in SOA is calculated as follows:

$$\text{Peroxide content (nmol µg}^{-1}) = \frac{\text{measured peroxide concentration (µM)} \times \text{reaction volume (mL)}}{\text{sample mass (µg)}} \quad \text{(S4)}$$

It is unlikely that the low peroxide contribution for naphthalene SOA is caused by the assumption we use to calculate peroxide contribution, i.e., peroxides present in the SOA possess the same DTT reactivity as benzoyl peroxide. Firstly, we measured the DTT reactivity for peroxides with different structures, including benzoyl peroxide, hydrogen peroxide, and cumene hydroperoxide. As shown in Figure S7B, there is no significant difference between the DTT reactivity of these peroxides. Secondly, it is known that quinones (e.g., 1,4-naphthoquinone) are major products formed from OH oxidation of naphthalene and they possess much higher DTT reactivity than those of certain peroxides (e.g., benzoyl peroxide and hydrogen peroxide).[12,18] Given the high abundance of quinones in naphthalene SOA and their high DTT reactivity, they have the potential to be a major OP contributor. Thirdly, a previous study on naphthalene SOA indicates that peroxides are not a major contributor to the OP,[16] which is consistent with the current measurement results. Regardless, further study is needed to more accurately estimate the contribution of peroxides to OP when the chemical standards

for more SOA-relevant peroxides are available. Regardless, this preliminary assessment of C_p here provides qualitative insights into the role of peroxides as a driver of the OP of the SOA.

C_p estimation

The contribution of peroxides to the OP of SOA (C_p; %) can be calculated using Eq. S5:

$$C_P = \frac{\text{Estiamted DTT}_{\text{peroxide}}}{\text{DTT}_{\text{SOA}}} \times 100\% \quad \text{(S5)}$$

DTT_{SOA} is the measured DTT decay rate (μM min^{-1}) during OP experiments, which can be calculated as follows:

$$\text{DTT}_{\text{SOA}} = \frac{\text{OP}_{\text{SOA}}\ (\text{pmol min}^{-1}\ \mu\text{g}^{-1}) \times \text{sample mass}\ (\mu\text{g})}{\text{reaction volume (mL)}} \quad \text{(S6)}$$

$DTT_{peroxide}$ is the predicted DTT decay rate (μM min^{-1}) due to SOA-bound peroxides during OP experiments, which can be calculated as follows.

Based on the measured peroxide content in SOA (see Eq. S4) and sample mass, the concentration of peroxide can be estimated:

$$\text{Peroxide concentration}\ (\mu\text{M}) = \frac{\text{peroxide content (nmol}\ \mu\text{g}^{-1}) \times \text{sample mass}\ (\mu\text{g})}{\text{reaction volume (mL)}} \quad \text{(S7)}$$

Given that most peroxides in SOA are unknown and lack standards, we assume that all peroxides have the same DTT reactivity as benzoyl peroxide. This assumption has been used in previous analysis of peroxides in polycyclic aromatic hydrocarbon-derived SOA,[16] due to the structural similarity between benzoyl peroxide and those formed during oxidation. The DTT reactivity of benzoyl peroxide is similar to those of other peroxides such as hydrogen peroxide (Figure S6B). With this assumption, the $DTT_{peroxide}$ can be calculated:

$$\text{Estimated DTT}_{\text{peroxide}}\ (\mu\text{M min}^{-1}) = \text{Peroxide concentration}\ (\mu\text{M}) \times \text{DTT reactivity for benzoyl peroxide} \quad \text{(S8)}$$

S5. Further perspectives

This is the first study to report the OP of oil sands SOA, which is subject to uncertainties and will require further investigation. Firstly, while this work focuses on the OP of SOA derived from oil sands ore, which represent an important emission source in the OS region, further study is needed to elucidate the potential health impacts of SOA derived from other emission sources such as tailing ponds, and/or the processing of bituminous ore within oil sands facilities. Secondly, the experiments were conducted under low NO_x conditions. However, it can be expected that SOA composition will be different under high NO_x conditions. For example, it has been shown that OH oxidation of oil sands-related precursors in the presence of NO_x generally forms less SOA mass as a result of the formation of higher volatility compounds. A

previous study has investigated the impact of NO_x on the OP of SOA formed from four different precursors (toluene, α-pinene, 1,3,5-trimethylbenzene, and isoprene). A lower OP was observed for isoprene SOA under high NO_x conditions, while no significant impacts were observed for the OP of other types of SOA. This highlights the need to investigate the OP of oil sands-related SOA formed in the presence of NO_x. Thirdly, the composition of ambient particles in OS regions may be more complex than the lab-generated oil sands SOA. For example, synergistic and/or antagonistic effects may occur during the interactions between transition metals (may be present in ambient particles) and organics in ambient particles. A comprehensive understanding of the PM exposure risk in local communities requires the analysis of the OP of ambient particles in OS regions.

S6. Modelled OH in oil sands plumes for OFR photochemical age determination

In order to estimate the average OH concentration in Oil Sands plumes, we simulated 20 days in the summer of 2018. The 20 days were re-run out of a longer model run in order to save additional diagnostic variables, including OH, which were not saved from the longer run. Figure S1A shows a histogram of the OH concentrations at 19H UTC (12:00 local time) while Figure S1B shows a map of the average concentration across the 20 days at 19H UTC (12:00 local time) for the lowest model layer (approximately 0-40m above the surface).

The simulations were conducted using GEM-MACH, ECCC's online weather and air quality model.[19-22] The model used in these simulations was based on GEM-MACH v3.1.0b.2 with several updates related to improvements to processes relevant to the Alberta Oil Sands Region (AOSR). These updates included: updates to the gas-phase chemistry mechanism to SAPRC11, [23] and the addition of a new inorganic heterogenous chemistry scheme,[24] a new plume rise formulation which accounts for the latent heat released by condensation of stack exhaust water vapor,[19] updated cloud scavenging,[25] and online photolysis.[26] Relevant to this study, SAPRC11 includes 20 additional reactions involving OH, including photolytic production from HONO and H_2O_2. The model uses a terrain following hybrid coordinate system with 84 levels. The levels are more compressed in the lower troposphere with the lowest model layer being approximately 40 m thick (although this varies throughout the modelling domain and through time). The model uses semi-Lagrangian advection[27] and finite-difference vertical diffusion with area source emissions applied as a lower boundary condition on the diffusion equation. Emissions were taken from the National Pollutant Release Inventory (NPRI) and the Air Pollutant Emission Inventory (APEI) from the year 2015 and scaled to the year 2018, with additional modifications to show better agreement with aircraft and surface observations from 2018.

S7. Further discussion on SOA composition and OP of SOA

Our previous study indicated that the formation yields of SOA from precursors such as α-pinene, decane, and oil sands ore increased at low photochemical ages and then remained at a relatively stable level at high photochemical ages (experiments were conducted using the same OFR system).[1] Given this, the change of OP is related to SOA composition rather than SOA mass.

For the decane reaction system, we did not find evidence for the formation of particulate quinones and unsaturated carbonyls from EESI-TOF-MS measurements. This is expected given the structure of decane. Previous studies on alkanes suggest that they may produce a very small fraction of unsaturated carbonyls (i.e., not a major product) and cannot form quinones during oxidation.[28]

For *m*-xylene reaction system, we also did not observe the mass signals of the potentially formed quinones. It is known that the formation yields of quinones from OH oxidation of single-ring aromatics (toluene and *m*-xylene) are very low.[29,30] As a result, the signals of the potentially formed quinones may be below the detection limit of EESI-TOF-MS. A potential unsaturated carbonyl product formed from *m*-xylene photooxidtion is $C_7H_8O_3$ (see Figure S4). The evolution of $C_7H_8O_3$ is shown in Figure S6, which is well correlated with the observed OP across the investigated photochemical ages.

For oil sand ore reaction system, given the chemical nature of OS ore off-gassing (a complex mixture of aromatics, alkanes, and alkenes), it is difficult to infer the structures of oxidation products present in oil sands SOA based on their mass signals only. Regardless, we notice that $C_{12}H_{22}O$ (a potential unsaturated carbonyl) is formed during photooxidation and its evolution pattern is shown in Figure S6.

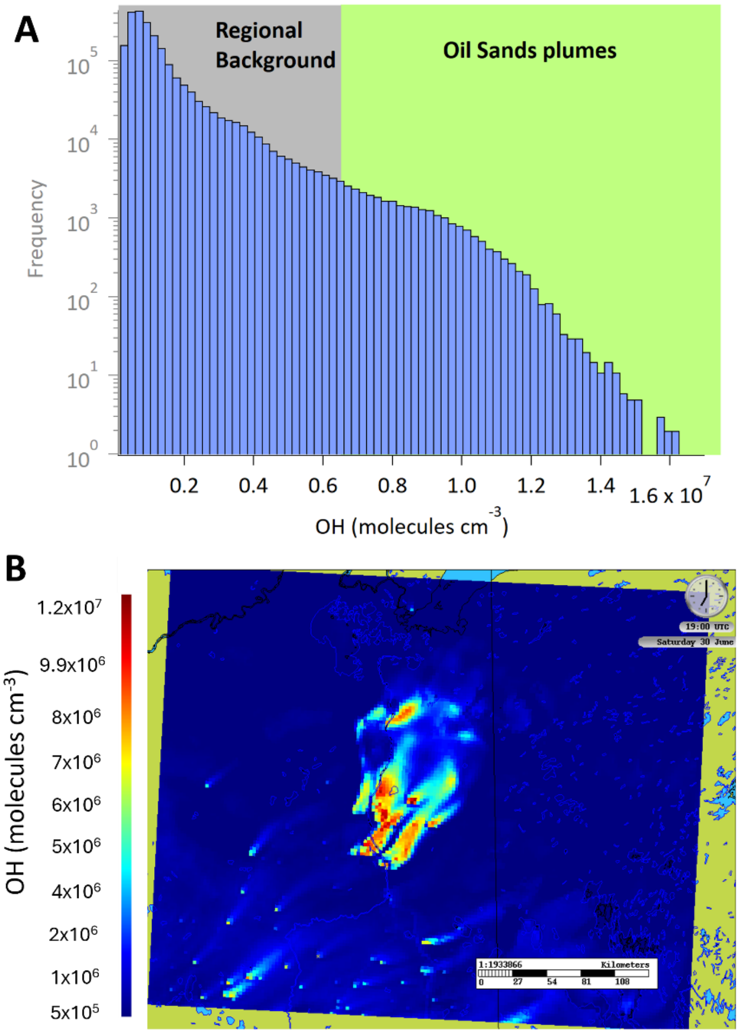

Figure S1. (**A**) Modelled OH concentration histogram for the oil sands domain, from the surface to 140 m altitude for 20 days in June 2018 (19:00 UTC). (**B**) Oil sands plumes consistently are highly oxidative as they age, with OH values >1 ×10^7 molecules cm^{-3}. See Text S6 for additional details.

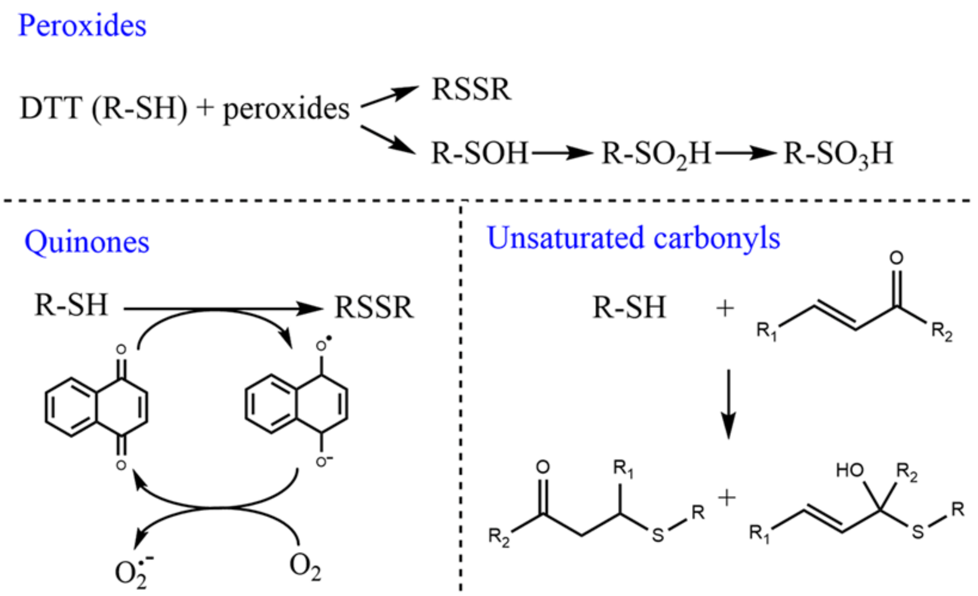

Figure S2. Reaction mechanism between DTT and organics in SOA (peroxides, quinones, and unsaturated carbonyls).[16,31]

Formation mechanism of peroxides

$RH + \cdot OH + O_2 \rightarrow R'\ CH_2OO\cdot + H_2O$

$R'CH_2OO\cdot + HO_2\cdot \rightarrow R'CH_2OOH + O_2$

Degradation mechanism of peroxides

(1) Photolysis reaction

$ROOH \rightarrow RO\cdot + \cdot OH$; $ROOH \rightarrow RO_2\cdot + H\cdot$; $ROOH \rightarrow R\cdot + HO_2\cdot$

(2) Reaction with carbonyls

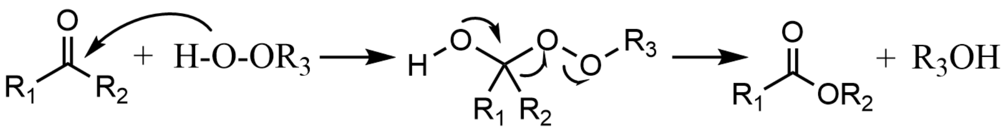

Figure S3. Formation and degradation mechanisms for peroxides.[32,33]

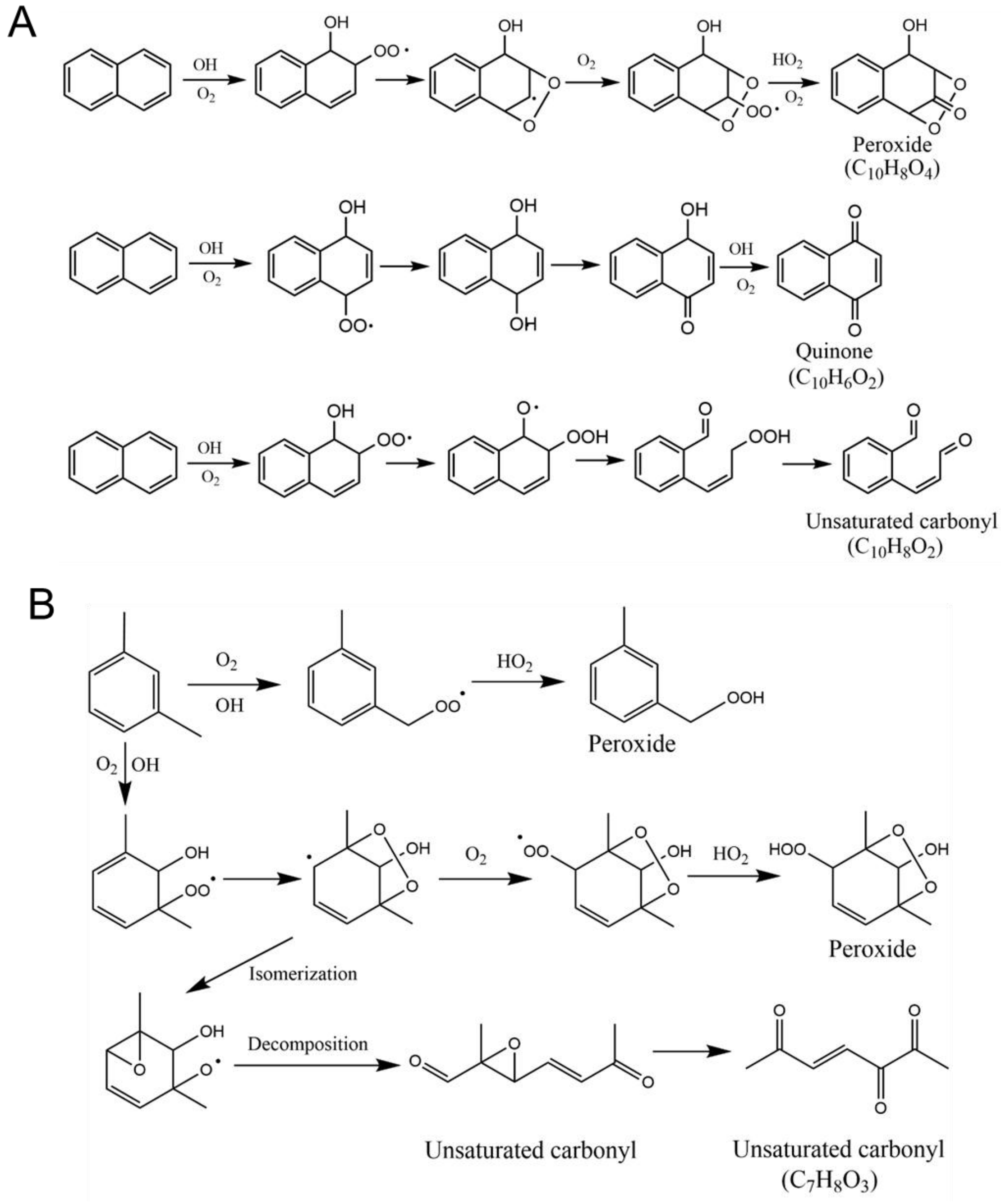

Figure S4. (A) General formation mechanism of peroxides, quinones, and unsaturated carbonyls during OH oxidation of naphthalene[17,34] (B) General formation mechanism of peroxides and unsaturated carbonyls during OH oxidation of *m*-xylene.[35]

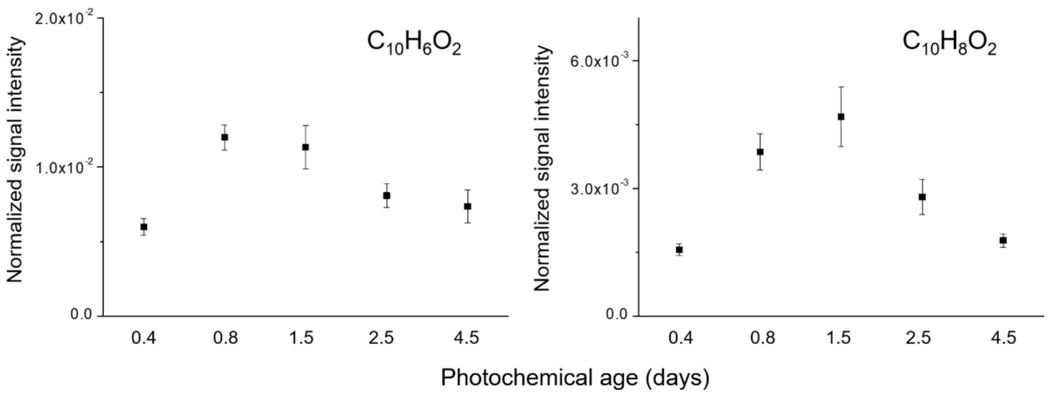

Figure S5. Normalized signal intensity of $C_{10}H_6O_2$ (likely attributed to naphthoquinones; measured with an EESI-TOFMS) and $C_{10}H_8O_2$ (likely attributed to an unsaturated carbonyl) in naphthalene SOA at different photochemical ages. Signals are normalized to the total signal intensity of $C_xH_yO_z$ (representing signals of organics) in EESI-TOFMS. Formation mechanism of naphthoquinones and unsaturated carbonyls is given in Figure S4.

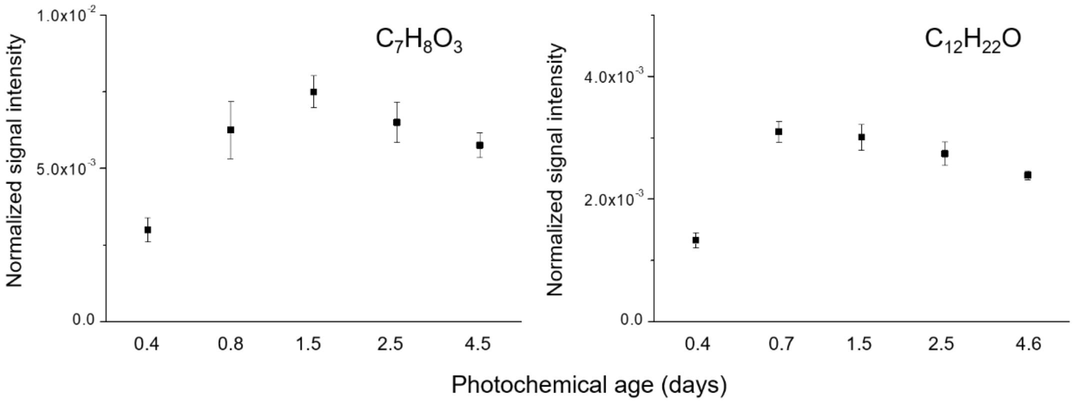

Figure S6. Normalized signal intensity of $C_7H_8O_3$ (a potential unsaturated carbonyl; measured with an EESI-TOFMS) in *m*-xylene SOA. Normalized signal intensity of $C_{12}H_{22}O$ (a potential unsaturated carbonyl; measured with an EESI-TOFMS) in oil sands ore SOA.

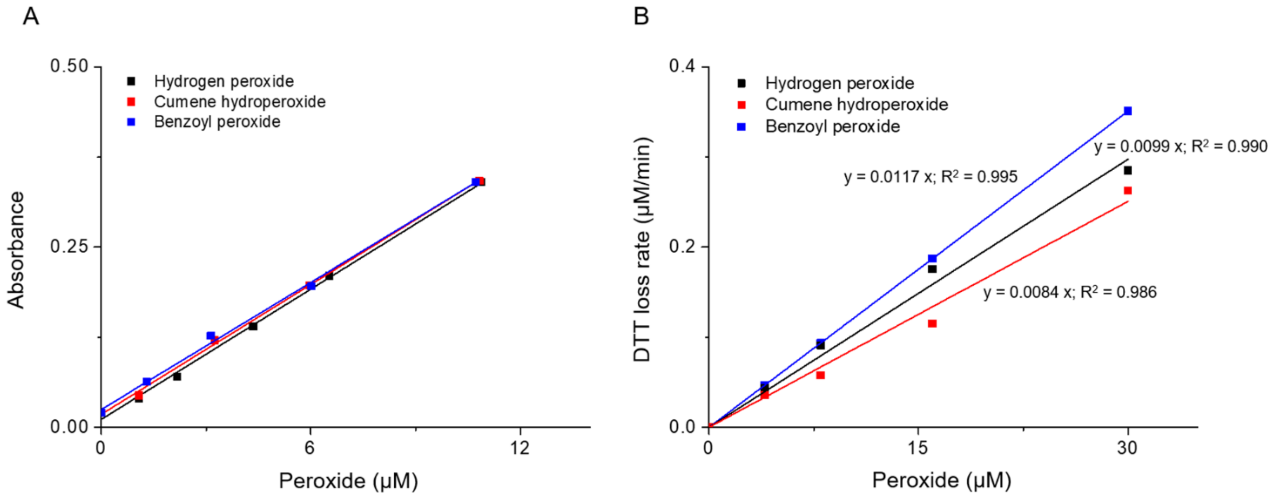

Figure S7. (A) Calibration curves for hydrogen peroxide, cumene hydroperoxide, and benzoyl peroxide. (B) DTT loss rate (μM/min) as a function of peroxide concentration (μM).

References

(1) Li, K.; Liggio, J.; Lee, P.; Han, C.; Liu, Q.; Li, S.-M. Secondary organic aerosol formation from α-pinene, alkanes, and oil-sands-related precursors in a new oxidation flow reactor. *Atmos. Chem. Phys.* **2019**, *19* (15), 9715-9731.
(2) He, M.; Ditto, J. C.; Gardner, L.; Machesky, J.; Hass-Mitchell, T. N.; Chen, C.; Khare, P.; Sahin, B.; Fortner, J. D.; Plata, D. L. Total organic carbon measurements reveal major gaps in petrochemical emissions reporting. *Science* **2024**, *383* (6681), 426-432.
(3) Lambe, A.; Chhabra, P.; Onasch, T.; Brune, W.; Hunter, J.; Kroll, J.; Cummings, M.; Brogan, J.; Parmar, Y.; Worsnop, D. Effect of oxidant concentration, exposure time, and seed particles on secondary organic aerosol chemical composition and yield. *Atmos. Chem. Phys.* **2015**, *15* (6), 3063-3075.
(4) Lopez-Hilfiker, F. D.; Pospisilova, V.; Huang, W.; Kalberer, M.; Mohr, C.; Stefenelli, G.; Thornton, J. A.; Baltensperger, U.; Prevot, A. S.; Slowik, J. G. An extractive electrospray ionization time-of-flight mass spectrometer (EESI-TOF) for online measurement of atmospheric aerosol particles. *Atmos. Meas. Tech.* **2019**, *12* (9), 4867-4886.
(5) Liu, Q.; Liggio, J.; Wentzell, J.; Lee, P.; Li, K.; Li, S.-M. Atmospheric OH Oxidation Chemistry of Particulate Liquid Crystal Monomers: An Emerging Persistent Organic Pollutant in Air. *Environ. Sci. Technol. Lett.* **2020**, *7* (9), 646-652.
(6) Liu, Y.; Sander, S. P. Rate constant for the OH + CO reaction at low temperatures. *J. Phys. Chem. A* **2015**, *119* (39), 10060-10066.
(7) Liggio, J.; Makar, P.; Li, S.-M.; Hayden, K.; Darlington, A.; Moussa, S.; Wren, S.; Staebler, R.; Wentzell, J.; Wheeler, M. Organic carbon dry deposition outpaces atmospheric processing with unaccounted implications for air quality and freshwater ecosystems. *Sci. Adv.* **2025**, *11* (1), eadr0259.
(8) Sheu, R.; Marcotte, A.; Khare, P.; Charan, S.; Ditto, J. C.; Gentner, D. R. Advances in offline approaches for chemically speciated measurements of trace gas-phase organic compounds via adsorbent tubes in an integrated sampling-to-analysis system. *J. Chromatogr. A* **2018**, *1575*, 80-90.
(9) Khare, P.; Marcotte, A.; Sheu, R.; Walsh, A. N.; Ditto, J. C.; Gentner, D. R. Advances in offline approaches for trace measurements of complex organic compound mixtures via soft ionization and high-resolution tandem mass spectrometry. *J. Chromatogr. A* **2019**, *1598*, 163-174.
(10) Bates, J. T.; Fang, T.; Verma, V.; Zeng, L.; Weber, R. J.; Tolbert, P. E.; Abrams, J. Y.; Sarnat, S. E.; Klein, M.; Mulholland, J. A. Review of acellular assays of ambient particulate matter oxidative potential: Methods and relationships with composition, sources, and health effects. *Environ. Sci. Technol.* **2019**, *53* (8), 4003-4019.
(11) Tuet, W. Y.; Chen, Y.; Xu, L.; Fok, S.; Gao, D.; Weber, R. J.; Ng, N. L. Chemical oxidative potential of secondary organic aerosol (SOA) generated from the photooxidation of biogenic and anthropogenic volatile organic compounds. *Atmos. Chem. Phys.* **2017**, *17* (2), 839-853.
(12) Jiang, H.; Jang, M.; Sabo-Attwood, T.; Robinson, S. E. Oxidative potential of secondary organic aerosols produced from photooxidation of different hydrocarbons using outdoor chamber under ambient sunlight. *Atmos. Environ.* **2016**, *131*, 382-389.

(13) Kramer, A. J.; Rattanavaraha, W.; Zhang, Z.; Gold, A.; Surratt, J. D.; Lin, Y.-H. Assessing the oxidative potential of isoprene-derived epoxides and secondary organic aerosol. *Atmos. Environ.* **2016**, *130*, 211-218.
(14) Lin, M.; Yu, J. Z. Dithiothreitol (DTT) concentration effect and its implications on the applicability of DTT assay to evaluate the oxidative potential of atmospheric aerosol samples. *Environ. Pollut.* **2019**, *251*, 938-944.
(15) Mertes, P.; Pfaffenberger, L.; Dommen, J.; Kalberer, M.; Baltensperger, U. Development of a sensitive long path absorption photometer to quantify peroxides in aerosol particles (Peroxide-LOPAP). *Atmos. Meas. Tech.* **2012**, *5* (10), 2339-2348.
(16) Wang, S.; Ye, J.; Soong, R.; Wu, B.; Yu, L.; Simpson, A. J.; Chan, A. W. Relationship between chemical composition and oxidative potential of secondary organic aerosol from polycyclic aromatic hydrocarbons. *Atmos. Chem. Phys.* **2018**, *18* (6), 3987-4003.
(17) Kautzman, K.; Surratt, J.; Chan, M.; Chan, A.; Hersey, S.; Chhabra, P.; Dalleska, N.; Wennberg, P.; Flagan, R.; Seinfeld, J. Chemical composition of gas-and aerosol-phase products from the photooxidation of naphthalene. *J. Phys. Chem. A* **2010**, *114* (2), 913-934.
(18) McWhinney, R. D.; Badali, K.; Liggio, J.; Li, S.-M.; Abbatt, J. P. Filterable redox cycling activity: a comparison between diesel exhaust particles and secondary organic aerosol constituents. *Environ. Sci. Technol.* **2013**, *47* (7), 3362-3369.
(19) Fathi, S.; Makar, P.; Gong, W.; Zhang, J.; Hayden, K.; Gordon, M. The importance of moist thermodynamics on neutral buoyancy height for plumes from anthropogenic sources. *Atmos. Chem. Phys.* **2025**, *25* (4), 2385-2405.
(20) Makar, P. A.; Akingunola, A.; Aherne, J.; Cole, A. S.; Aklilu, Y.-a.; Zhang, J.; Wong, I.; Hayden, K.; Li, S.-M.; Kirk, J. Estimates of exceedances of critical loads for acidifying deposition in Alberta and Saskatchewan. *Atmos. Chem. Phys.* **2018**, *18* (13), 9897-9927.
(21) Moran, M.; Ménard, S.; Talbot, D.; Huang, P.; Makar, P.; Gong, W.; Landry, H.; Gravel, S.; Gong, S.; Crevier, L. Particulate-matter forecasting with GEM-MACH15, a new Canadian air-quality forecast model. *Steyn, DG and Rao, ST, Springer, Dordrecht* **2010**, 289-292.
(22) Moran, M. D.; Lupu, A.; Zhang, J.; Savic-Jovcic, V.; Gravel, S. A comprehensive performance evaluation of the next generation of the Canadian operational regional air quality deterministic prediction system. In *International Technical Meeting on Air Pollution Modelling and its Application*, 2016; Springer: pp 75-81.
(23) Carter, W. P.; Heo, G. Development of revised SAPRC aromatics mechanisms. *Atmos. Environ.* **2013**, *77*, 404-414.
(24) Miller, S. J.; Makar, P. A.; Lee, C. J. HETerogeneous vectorized or Parallel (HETPv1. 0): an updated inorganic heterogeneous chemistry solver for the metastable-state NH 4+–Na+–Ca 2+–K+–Mg 2+–SO 4 2-–NO 3-–Cl-–H 2 O system based on ISORROPIA II. *Geoscientific Model Development* **2024**, *17* (6), 2197-2219.
(25) Ghahreman, R.; Gong, W.; Makar, P. A.; Lupu, A.; Cole, A.; Banwait, K.; Lee, C.; Akingunola, A. Modeling below-cloud scavenging of size-resolved particles in GEM-MACHv3. 1. *Geoscientific Model Development* **2024**, *17* (2), 685-707.
(26) Majdzadeh, M.; Stroud, C. A.; Sioris, C.; Makar, P. A.; Akingunola, A.; McLinden, C.; Zhao, X.; Moran, M. D.; Abboud, I.; Chen, J. Development of aerosol optical properties for improving the MESSy photolysis module in the GEM-MACH v2. 4 air quality model and

application for calculating photolysis rates in a biomass burning plume. *Geoscientific Model Development* **2022**, *15* (1), 219-249.
(27) Yeh, K.-S.; Côté, J.; Gravel, S.; Méthot, A.; Patoine, A.; Roch, M.; Staniforth, A. The CMC–MRB global environmental multiscale (GEM) model. Part III: Nonhydrostatic formulation. *Monthly Weather Review* **2002**, *130* (2), 339-356.
(28) Schilling Fahnestock, K. A.; Yee, L. D.; Loza, C. L.; Coggon, M. M.; Schwantes, R.; Zhang, X.; Dalleska, N. F.; Seinfeld, J. H. Secondary organic aerosol composition from C12 alkanes. *J. Phys. Chem. A* **2014**, *119* (19), 4281-4297.
(29) Hamilton, J. F.; Webb, P. J.; Lewis, A. C.; Reviejo, M. M. Quantifying small molecules in secondary organic aerosol formed during the photo-oxidation of toluene with hydroxyl radicals. *Atmos. Environ.* **2005**, *39* (38), 7263-7275.
(30) Li, Y.; Zhao, J.; Gomez-Hernandez, M.; Zhang, R. Functionality-based formation of secondary organic aerosol from m-xylene photooxidation. *Atmospheric Chemistry and Physics Discussions* **2021**, *2021*, 1-27.
(31) Jiang, H.; Jang, M.; Yu, Z. Dithiothreitol activity by particulate oxidizers of SOA produced from photooxidation of hydrocarbons under varied NO x levels. *Atmos. Chem. Phys.* **2017**, *17* (16), 9965-9977.
(32) Hallquist, M.; Wenger, J. C.; Baltensperger, U.; Rudich, Y.; Simpson, D.; Claeys, M.; Dommen, J.; Donahue, N.; George, C.; Goldstein, A. The formation, properties and impact of secondary organic aerosol: current and emerging issues. *Atmos. Chem. Phys.* **2009**, *9* (14), 5155-5236.
(33) Saunders, S. M.; Jenkin, M. E.; Derwent, R. G.; Pilling, M. J. Protocol for the development of the Master Chemical Mechanism, MCM v3 (Part A): tropospheric degradation of non-aromatic volatile organic compounds. *Atmos. Chem. Phys.* **2003**, *3* (1), 161-180.
(34) Keyte, I. J.; Harrison, R. M.; Lammel, G. Chemical reactivity and long-range transport potential of polycyclic aromatic hydrocarbons–a review. *Chem. Soc. Rev.* **2013**, *42* (24), 9333-9391.
(35) Zhang, Q.; Xu, Y.; Jia, L. Secondary organic aerosol formation from OH-initiated oxidation of m-xylene: effects of relative humidity on yield and chemical composition. *Atmos. Chem. Phys.* **2019**, *19* (23), 15007-15021.
